# Trauma Care in Oman: A Narrative Review on Current Practices, Challenges, and Developmental Priorities

**DOI:** 10.7759/cureus.97783

**Published:** 2025-11-25

**Authors:** Ruqaiya Al-Habsi

**Affiliations:** 1 Surgery, The Royal London Hospital, London, GBR

**Keywords:** major trauma, oman, sultanate of oman, trauma and orthopedics, trauma centre

## Abstract

Having an effective trauma system is essential for reducing avoidable morbidity and mortality. The Sultanate of Oman, like many countries, faces a significant burden of trauma cases presenting either directly to emergency centers, private healthcare facilities, or through emergency services seeking urgent care. Despite major advances in infrastructure and healthcare over recent decades, trauma remains a major challenge, particularly due to variation in trauma facilities and healthcare access across different regions of the country. This narrative review examines the current state of trauma care in Oman and aims to identify both strengths and challenges within the healthcare system related to trauma management. Addressing these challenges, and ensuring they are incorporated into Oman’s Vision 2040 agenda, is vital to reducing preventable morbidity and mortality and improving the overall effectiveness of the national trauma care system.

## Introduction and background

Trauma remains a major global health challenge, despite being largely preventable. Road traffic accidents (RTAs) have been identified by the World Health Organization (WHO) as a major public health concern, with more than a million deaths annually. The “Golden Hour” is a key factor in reducing RTA-related mortality, emphasizing the importance of transporting patients to a trauma facility within 60 minutes of injury [1]. Many trauma victims die before reaching a hospital, likely due to underdeveloped pre-hospital care and transport systems. Ensuring timely and appropriate emergency care can be life-changing and, in many cases, life-saving. Establishing an effective emergency medical service (EMS) remains a major challenge for both developing and developed countries, including the Sultanate of Oman [2]. Trauma systems are an essential component of healthcare, as they influence patient outcomes and ensure timely access to specialized care [3].

The Sultanate of Oman is located on the southeastern coast of the Arabian Peninsula and is one of the six member states of the Gulf Cooperation Council (GCC), along with Bahrain, Kuwait, Qatar, the Kingdom of Saudi Arabia (KSA), and the United Arab Emirates (UAE) [4]. According to the 2025 World Population Review, Oman’s population has reached 5.5 million, with a density of 17.75 per km² and an annual growth rate of 213.2 thousand (3.97%) [5]. More than two-thirds of the population resides in urban areas, with over half living in the Muscat governorate and the Batinah coastal plain northwest of the capital [4]. The 2023/2024 United Nations Human Development Report ranks Oman as a very highly developed country, with a Human Development Index of 0.819 [6]. 

Trauma and related injuries are among the leading causes of mortality in Omani hospitals and are a major contributor to disability-adjusted life years. Age-standardized mortality from trauma is 53 per 100,000 population [7]. RTAs are the most common cause of trauma seen in hospitals. Some studies have reported that Omani trauma patients lived an average of 7.2 years with disability per 100 individuals, although the specific types of disabilities were not clearly identified. Unfortunately, many of these studies are outdated and may not reflect current data from the past five years [8]. 

Trauma affects Omani males disproportionately, and studies show wide geographic variation in trauma incidence. This does not align with the distribution of trauma care capabilities, as most high-level healthcare resources are concentrated in the northern region of the country. Although many regions have hospitals and primary care centers, higher-quality care and access to specialized trauma surgeons remain primarily available near the capital. Therefore, equitable distribution of trauma services is essential to improve emergency care access and prevent avoidable mortality and morbidity due to delayed treatment [9]. 

This narrative review aims to provide an overview of Oman’s current trauma care system, with a focus on pre-hospital emergency services, post-trauma rehabilitation, and the national trauma registry. The goal is to offer evidence-based recommendations that can contribute to existing knowledge and support improvements in the Omani healthcare system by evaluating published data.

## Review

Methods 

This narrative review examines the current trauma system in the Sultanate of Oman, the challenges it faces, and recommendations for future development. Evidence was collected from EMBASE, PubMed, and Google Scholar up to October 2025. In addition to database searches, information was gathered from government websites, including the Ministry of Health and the Civil Defence and Ambulance Authority of Oman. The review and writing process took place between June 2025 and October 2025.

Search Terms, Inclusion Criteria, and Exclusion Criteria

The search terms used to identify relevant papers included “Oman”, “Sultanate of Oman”, “Trauma”, “Trauma system”, “Ministry of Health Oman”, “Ambulance Oman”, “Oman Trauma Registry”, “WHO Oman Trauma”, “Middle East”, “Trauma care Oman”, and “Motor Vehicle Accident.”

The study inclusion period was not restricted to a specific timeframe, although more recent publications were prioritized. Because only a limited number of papers on trauma and trauma systems in Oman were available, most studies meeting these criteria were included.

All included studies involved human subjects across all age groups and genders.

All animal studies were excluded.

Burden and epidemiology of trauma in Oman 

Globally, Oman is ranked among the countries with the highest rates of RTAs. In 2015 alone, there were 6,279 RTAs, resulting in 2,624 injuries and 675 deaths [10].

Jeswani et al. published a paper in the Oman Medical Journal (OMJ) in 2025, presenting a retrospective chart review of pediatric patients aged up to 15 years who presented to the accident and emergency department between January and December 2022 [11]. They identified 1,643 patients who met the inclusion criteria. The most common cause of injury was a fall from height (50.8%), followed by collisions with fixed objects and sports-related injuries. Adolescents had higher rates of sports-related injuries and RTAs, some requiring admission to intensive care units, especially those with polytrauma from road traffic accidents [11].

Assault-induced trauma (AIT) can be life-threatening and often requires a multidisciplinary approach. Injury severity ranges from morbidity, such as scarring, prolonged hospitalization, and limb loss, to mortality if not managed promptly. According to the 2016 Omani statistics, 1,631 assault cases were reported in 2015. However, up-to-date literature on AIT in Oman remains limited [12].

In 2013, the Ministry of Health (MOH) of Oman reported 646 deaths on arrival (DOAs) related to RTAs, occurring either before reaching the hospital or upon arrival. Additionally, in the same year, 68.4% of all inpatient mortalities were attributed to RTAs [1].

In 2018, a retrospective study assessed the incidence of trauma among all patients aged 0-15 years between November 2014 and April 2015 at two Omani hospitals (Khoula and Nizwa). The study included all patients who triggered a trauma team activation in the emergency department. A total of 795 pediatric trauma cases were identified (>30% of all injury admissions), with 92% classified as minor injuries (Injury Severity Score (ISS) < 9). Most of these injuries were due to unintentional mechanisms [13].

Current trauma care delivery

Given the ongoing burden of RTAs as a major cause of injury in Oman, the limited data on trauma-related disabilities, and the high rates of injury and injury-related mortality, it is essential for the country to establish well-structured and well-documented trauma systems. Such systems ensure timely and appropriate management of trauma patients and provide a reliable trauma database to support continuous quality improvement.

Trauma cases require close monitoring and are often time-critical, particularly in patients with active bleeding or multiple organ injuries [14]. The composition of a trauma team varies depending on injury location and severity, but typically includes trauma and orthopedic surgeons, general surgeons, neurosurgeons, radiologists, radiographers, and a trauma lead, usually an emergency medicine physician.

Since the ascension of Sultan Qaboos bin Said (may he rest in peace) in 1970, healthcare administration in Oman has been overseen by the Ministry of Health (MOH), operating under the Directorate General of Health Affairs. The MOH is responsible for planning and implementing national health policies and programs, with support from governorate-level health authorities [9].

Oman currently has a three-tiered healthcare system comprising primary care (primary healthcare centers providing acute and chronic care), secondary care (regional referral hospitals), and tertiary care (specialized referral hospitals). In terms of trauma care, initial management, including patient resuscitation and stabilization, occurs in primary health centers, which then refer patients to secondary or tertiary hospitals as needed [15]. These facilities are distributed throughout the country, with primary health centers being the most widespread. Secondary hospitals are generally located in larger towns (wilayat), while tertiary and referral hospitals are concentrated in the capital, Muscat. These higher-level hospitals are run by the MOH as well as the Ministry of Defence (MOD), Royal Oman Police (ROP), Petroleum Development Oman (PDO), and Sultan Qaboos University (SQU) [9].

Pre-hospital Care

In 2000, the need for ambulance services in Oman was formally recognized, leading to the planning of an Ambulance Project supported by the Civil Defence Law No. 91/76 and ROP Law No. 90/35. Both were later amended through a Royal Decree, and in 2001, an Ambulance Division Project Committee was established to move the initiative forward [16].

The trauma system in Oman includes the EMS and the pre-hospital emergency care system, both of which are operated by the Public Authority of Civil Defence and Ambulances. Hospitals across the country vary greatly in size and capability: some are tertiary hospitals with trained trauma and non-trauma surgeons, while others are small rural health centers mainly staffed by junior, non-specialist doctors [10].

Oman’s EMS officially began operations in April 2004 and employed well-trained Omani advanced emergency medical technicians (AEMTs), who were trained locally by Omani instructors [17]. Paramedics are currently stationed across 36 centers throughout the country [18], and their scope of practice covers all medical emergencies, including trauma. Most of their workload consists of trauma cases (83%), with the remaining 17% related to medical emergencies. However, there is no published data assessing the system’s effectiveness in reducing morbidity and mortality from RTAs [17].

Omani AEMTs follow seven core values in their practice: honesty, respect, compassion, excellence, teamwork, accountability, and foresight to anticipate the needs of their communities [16].

Hospital-Based Emergency Care 

Al-Kindi et al. published a pilot study in 2018 that reviewed the distribution of trauma care facilities in Oman in relation to RTAs [1]. They identified 32 trauma care facilities and assessed their classification according to the American College of Surgeons Committee on Trauma (ACS COT). Table 1 outlines the ACS COT trauma center classifications. In Oman, four centers were classified as level V facilities, located across three governorates, including Muscat. The authors also examined the distance between trauma facilities and known RTA “hotspot” areas. They found an average distance of 34.7 km between hotspots and the nearest trauma facility, while the average distance to level IV or V facilities was notably higher at 83.3 km [1].

**Table 1:** The American College of Surgeons Committee on Trauma (ACS COT) classification of trauma facilities Adapted from: [19] (Creative Commons Attribution-NonCommercial-NoDerivatives 4.0 International (CC BY-NC-ND 4.0)). ATLS: advanced trauma life support.

Classification level	Description
I	Tertiary care hospitals; able to provide extensive management from initial injury to rehabilitation
II	Provides initial assessment and management of all trauma patients; able to stabilize patients and transfer to facilities with more specialized care
III	Provides initial assessment and management, surgical management, and stabilization post-trauma
IV	Initial assessment using ATLS, and then transfer of the patient to a higher-care level facility
V	Initial assessment, and then coordinate patient transfer if advanced care is needed

The Sultan Qaboos University Hospital (SQUH) is a major teaching hospital in Oman with more than 600 beds and a wide catchment area. Its 25-bed accident and emergency department receives over 60,000 patients annually [11]. SQUH is the only tertiary hospital in the country with board-certified trauma surgeons trained in trauma, critical care, and acute care surgery. The emergency department manages approximately 900 trauma cases each year [10]. Based on the ACS criteria, SQUH functions as a level II trauma facility. Another key hospital in the region is the Khoula Hospital, which aligns with level III criteria, in addition to several lower-level centers across the country. This distribution underscores the need for a designated level I trauma center to ensure optimal care for severely injured patients [18].

Management of severe trauma often requires interventional radiology for hemostasis [14]. Patients are triaged using the Canadian Triage system and assessed with the ISS, ATLS protocols, and the Glasgow Coma Scale (GCS) [11].

Oman, similar to other Gulf countries, has a large expatriate population. Expatriates have access to emergency departments and receive trauma care free of charge, and many also have employer-sponsored insurance for non-emergency conditions [9].

Workforce, education, and training****

ATLS was first established by Dr. James Styner, a pilot who tragically lost his wife in a plane crash in 1976. His two children were seriously injured and taken to a local hospital, where the inadequate standard of care prompted him to develop a structured trauma system aimed at improving trauma management in rural areas [20].

ATLS was introduced in Oman in 2011, and by 2018, more than 378 providers had completed the training. This course is essential, and without formal ATLS training, trauma personnel may not be adequately prepared to deliver appropriate care. ATLS should be made mandatory for all physicians involved in the initial management and resuscitation of trauma victims in Oman [18].

Omani EMTs receive training through internationally recognized programs, including Basic Trauma Life Support (BTLS), ATLS, Advanced Cardiac Life Support (ACLS), Paediatric Advanced Life Support (PALS), and Pre-Hospital Trauma Life Support (PHTLS), among others. These courses provide emergency care providers with the necessary knowledge to manage a wide range of emergencies, from complex trauma to cardiac arrest [16].

In 2017, Al-Mahrouqi et al. conducted a retrospective review published in the *Oman Journal of Ophthalmology*, examining ocular trauma cases at Al-Nahdha Hospital over a six-month period (January-June 2013) [21]. They reported that 27,951 trauma patients, both ocular and non-ocular, presented to the emergency department, with males at higher risk of injury. This was the first epidemiologic study on ocular trauma in Oman and found that blunt objects, sharp tools, and nails were among the most common causes [21]. The study did not assess pre-hospital care, transport, or general hospital trauma management beyond ophthalmology. It highlighted the need for improved public health education on preventive measures, such as protective eyewear, and emphasized the importance of seeking appropriate trauma care [21].

These findings reinforce the need for comprehensive trauma training and a strong emphasis on both pre-hospital and in-hospital trauma care to reduce preventable morbidity and mortality in Oman.

Trauma registry****

The Trauma Audit and Research Network (TARN) is a major trauma registry used in the United Kingdom and has since been replaced by the National Major Trauma Registry. It remains the largest trauma registry in Europe [22]. TARN collects data on all trauma patients admitted to hospitals in England and Wales whose hospital stay exceeded three days, required critical care, required transfer to another facility for further treatment, or resulted in death due to their injuries [23]. Information collected includes patient demographics, injury characteristics and mechanisms, affected body regions, and assessment findings such as the ISS and GCS. Operative details are also included, along with the specialties involved in patient care [23]. Similar trauma registries exist worldwide. In the United States, the equivalent is the National Trauma Data Bank (NTDB), which includes the Trauma Quality Improvement Program (TQIP). In Australia, the counterpart is the Victorian State Trauma Outcomes Registry and Monitoring Group (VSTORM). These registries were established to support integrated, regional, multidisciplinary trauma systems that aim to deliver optimal care for major trauma patients [24].

Oman currently lacks a well-established national trauma registry. A pilot study published in 2017 in Global Health Action examined the feasibility of developing such a system by collecting data in two large hospitals in Oman. The study gathered information on more than 2,500 trauma patients, including demographics, injury sites, and relevant clinical details. The team also met with the Ministry of Health Information Technology Department to discuss further development. The authors concluded that this pilot study could serve as the foundation for a future national trauma registry in Oman [7].

Comparative insights****

Oman is part of the GCC region, which also includes Bahrain, Kuwait, Qatar, KSA, and the UAE. The combined GCC population was projected to increase from 56.4 million in 2018 to nearly 66 million by 2023. In response to this rapid growth, GCC countries have highlighted the need to adopt best practices in trauma system development. KSA’s Vision 2030 includes major upgrades to trauma care, with significant investment in emergency services. Oman and Qatar launched Vision 2040, both emphasizing the principle of “Health for All.” Kuwait and Bahrain have likewise stressed the need for EMS modernization, the establishment of trauma registries, and the implementation of coordinated national guidelines and policies [24].

Qatar has made substantial progress in trauma care. Its national trauma system received the Trauma Distinction Award from Accreditation Canada International in 2014, the first such recognition in the Middle East. That same year, the Hamad Trauma Unit was established at Hamad General Hospital, providing multidisciplinary trauma care with trauma surgeons, emergency physicians, anesthetists, and ICU nurses [25].

The UAE and KSA both share borders with Oman and have more developed EMS systems. In the UAE, EMS services are divided between Dubai EMS, which is regulated by the police, and the National Ambulance, which serves the remaining Emirates. The system operates more than 110 ambulances across over 50 ambulance stations, in addition to one medical helicopter. Their national response time, however, has not been reported. KSA’s EMS is able to respond within 10-13 minutes and is supported by 1,379 ambulances, 14 helicopters, and 486 dispatch centers [19]. Beyond pre-hospital care, KSA has also established the Saudi TraumA Registry (STAR), which became fully operational in 2018 [26].

Challenges, system gaps, and recommendations 

Upon reviewing the literature on trauma care and the emergency system in Oman, it appears that one of the major challenges facing the Omani EMS system is the country’s large geographical area, which can cause delays in reaching patients in a timely manner. Although Oman has already invested in and established more than 23 permanent ambulance stations across the region, further expansion is needed to ensure that even the most rural areas can be accessed quickly when required.

Another challenge is the absence of unified national guidelines for trauma care. Unlike the British Orthopaedic Association Standards for Trauma (BOAST) or the American Association for the Surgery of Trauma (AAST), Oman does not currently have a national framework to guide trauma management. Such guidelines are crucial to promote a standardized approach, reduce missed injuries, ensure timely diagnostics, and streamline appropriate management. Additionally, Oman still lacks a level I trauma facility. Based on existing literature and examples from surrounding countries, the establishment of a level I center is strongly recommended, as it would significantly enhance trauma care within the country.

One of the primary recommendations is to focus on developing an integrated and cohesive trauma system, which is likely more impactful than concentrating on individual healthcare organizations. Given the country’s large surface area, ensuring rapid access for rural communities and timely transfer to the nearest trauma center could play a pivotal role in reducing morbidity and mortality.

Finally, developing a national trauma registry, similar to TARN, NTDB, or VSTORM, is essential for the continuous improvement of Oman’s trauma system. Having accessible, reliable data and the ability to compare outcomes related to patient management will be invaluable for guiding system development and improving care moving forward.

## Conclusions

Oman has a 2040 vision that includes significant improvements in healthcare. The country continues to develop its infrastructure and ensure that people within its borders receive appropriate healthcare. One of the main challenges in trauma care is Oman’s vast surface area, which results in long distances between RTA locations and the nearest trauma care facility. Although a well-functioning EMS system has been established, it still struggles to cover the country’s wide geographic spread fully. In addition, Oman still needs an official trauma registry to record essential data and help evaluate and improve current practice. Because such a registry does not yet exist, there is limited published literature describing the structure of the Omani trauma system, the communication between EMS teams and trauma care facilities, or any unified national guidelines for initial trauma care. Developing an official, comprehensive trauma network supported by a well-maintained trauma registry is vital. This would allow for proper auditing, help identify what works and what needs improvement, and ultimately support the delivery of the best possible trauma care in the long term.

## References

[1] Al-Kindi SM, Naiem AA, Al-Gheiti KM (2018). Distribution of trauma care facilities in Oman in relation to high-incidence road traffic injury sites: pilot study. Sultan Qaboos Univ Med J.

[2] Mehmood A, Allen KA, Salami J, Kashmiri AA, Busaidi AA, Stevens K, Hyder AA (2016). 306 Pre-hospital care in Oman: results from hospital based trauma registry. Injury Prevention.

[3] Schipper IB, Simmen HP, Pfeifer R (2025). Trauma systems in Europe/hospital categories. Eur J Trauma Emerg Surg.

[4] Zahlan RS (2017). The Making of the Modern Gulf States: Kuwait, Bahrain, Qatar, the United Arab Emirates and Oman. routledge.

[5] (2025). World Population Review. Population of Oman. https://worldpopulationreview.com/countries/oman.

[6] Pedro Conceição (2024). Human Development Report 2023/2024. Breaking the Gridlock: Reimagining Cooperation in a Polarized World. United Nations Development Programme.

[7] Mehmood A, Chan E, Allen K (2017). Development of an mHealth trauma registry in the Middle East using an implementation science framework. Glob Health Action.

[8] Al-Adawi S, Al-Busaidi Z, Al-Adawi S, Burke DT (2012). Families coping with disability due to brain injury in Oman: attribution to belief in spirit infestation and ensorcellment. Sage Open.

[9] Mehmood A, Allen KA, Al-Maniri A, Al-Kashmiri A, Al-Yazidi M, Hyder AA (2017). Trauma care in Oman: a call for action. Surgery.

[10] Al-Kashmiri A, Al-Shaqsi SZ, Hasan N, Mahmood Mahmood (2017). Outcomes of multi-trauma road traffic crashes at a tertiary hospital in Oman: does attendance by trauma surgeons versus non-trauma surgeons make a difference?. Sultan Qaboos Univ Med J.

[11] Jeswani NL, Iram S, Shalash FM, Faraz R, Zeidan H, Al Reesi A (2025). Epidemiology of pediatric trauma and its outcome presenting to an emergency department in a tertiary care hospital in Oman. Oman Med J.

[12] Al-Busaidi M, Al-Miskry H, Al-Zadjali A, Ilyas Ilyas, Al-Saidi F, Al-Qadhi H (2021). Overview of assault-induced trauma presenting to a trauma centre in Oman. Sultan Qaboos Univ Med J.

[13] Mehmood A, Agrawal P, Allen KA, Al-Kashmiri A, Al-Busaidi A, Hyder AA (2018). Childhood injuries in Oman: retrospective review of a multicentre trauma registry data. BMJ Paediatr Open.

[14] Matsumoto N, Yamamoto S, Endo I (2019). The efficacy of a trauma call system: challenges in managing severe trauma at a rural emergency center without full-time emergency physicians. Acute Med Surg.

[15] Al Balushi A, Al Belushi Z, Asma A (2022). Evaluating trauma care capabilities using the essential trauma care guidelines of the World Health Organization. Sultan Qaboos Univ Med J.

[16] Al-Shaqsi SZ (2009). EMS in the Sultanate of Oman. Resuscitation.

[17] Al-Shaqsi S, Al-Kashmiri A, Al-Hajri H, Al-Harthy A (2014). Emergency medical services versus private transport of trauma patients in the Sultanate of Oman: a retrospective audit at the Sultan Qaboos University Hospital. Emerg Med J.

[18] Al-Kashmiri A (2017). Trauma care in Oman: where do we stand and where should we be heading?. Sultan Qaboos Univ Med J.

[19] Southern AP, Celik DH (2025). EMS: trauma center designation. StatPearls [Internet].

[20] Mohammad A, Branicki F, Abu-Zidan FM (2014). Educational and clinical impact of advanced trauma life support (ATLS) courses: a systematic review. World J Surg.

[21] Al-Mahrouqi HH, Al-Harthi N, Al-Wahaibi M, Hanumantharayappa K (2017). Ocular trauma. A tertiary hospital experience from Oman. Oman J Ophthalmol.

[22] Edwards A (2015). Top 10 TARN research publications. Emerg Med J.

[23] Bouderba S, Lecky F, Soltana K (2023). Comparison of trauma care structures, processes and outcomes between the English National Health Service and Quebec, Canada. Can J Surg.

[24] Khan L, Karim A, Bey H (2025). Evaluating Gulf Cooperation Council Trauma Care Infrastructure: a scoping review of key components and gaps. World J Surg.

[25] Al-Thani H, El-Menyar A, Asim M (2019). Evolution of the Qatar Trauma System. The journey from inception to verification. J Emerg Trauma Shock.

[26] Ford JE, Alqahtani AS, Abuzinada SA, Cameron PA, Fitzgerald MC, Alenizi AS, Farjou D (2020). Experience gained from the implementation of the Saudi TraumA Registry (STAR). BMC Health Serv Res.

